# A Rapid Evidence Support System Assessment (RESSA) of health policymaking in Ireland – A Protocol

**DOI:** 10.12688/hrbopenres.14167.1

**Published:** 2025-06-23

**Authors:** Barbara Whelan, Marie Tierney, Nikita N Burke, KM Saif-Ur-Rahman, Caitriona Creely, Trudy Duffy, Catherine Gill, Mary Horgan, John N Lavis, Teresa Maguire, Mairead O'Driscoll, John O'Neill, Kerry Waddell, Declan Devane

**Affiliations:** 1 Evidence Synthesis Ireland & Cochrane Ireland, University of Galway, Galway, Ireland; 2 Centre for Health Research Methodology, School of Nursing and Midwifery, University of Galway, Galway, Ireland; 3 Health Research Board, Dublin, Ireland; 4 Evidence for Policy Unit, Department of Further and Higher Education, Research, Innovation and Science, Dublin, Ireland; 5 Department of Health, Dublin, Ireland; 6 University College Dublin School of Medicine, Dublin, Ireland; 7 McMaster Health Forum, McMaster University, ON, Canada; 8 Department of Health Research Methods, Evidence, and Impact, McMaster University, ON, Canada; 9 Health Research and Innovation Policy Unit, Department of Health, Dublin, Ireland

**Keywords:** Rapid Evidence Support System Assessment, RESSA, evidence-informed policymaking, evidence-support system, health policymaking

## Abstract

**Background:**

Evidence-informed policymaking promotes the use of the best available evidence in a systematic and transparent manner to guide policy decisions. It aims to ensure that policies are grounded in credible and relevant evidence while also considering factors such as feasibility, sustainability, equity, and stakeholder input. The Global Evidence Commission has emphasised the necessity for stronger national evidence infrastructures and recommended that governments evaluate their evidence-support systems, focusing on the demand for evidence from policymakers, the supply of timely and relevant evidence, and the coordination between the two. To assist countries in reviewing their evidence-support systems, the Global Commission on Evidence to Address Societal Challenges developed the Rapid Evidence Support System Assessment (RESSA). Here, we outline the protocol for a RESSA of health policymaking being conducted in Ireland.

**Methods:**

This study will adopt a flexible, mixed-methods design with four key stages: (1) a high-level website review, (2) an in-depth document review, (3) semi-structured interviews with key stakeholders, and (4) seeking feedback. For the document review, the data analysis and synthesis process will follow the READ approach, allowing for a systematic way to organise, interpret, and synthesise the information extracted from the selected documents. Interview data will be analysed using a thematic approach. Findings from both sources will be triangulated to ensure robust conclusions about the strengths and challenges of the evidence-support system for health policymaking.

**Conclusions:**

This protocol outlines the methods for assessing Ireland’s evidence support system for health policymaking. By documenting our approach in detail, we aim to enhance transparency and replicability, providing a foundation for easier comparison and contrast with similar assessments conducted by other groups. While this study focuses on health, the methodology and findings may also inform evidence-support systems in other sectors, such as climate and education.

## Introduction

Evidence-informed policymaking promotes the systematic and transparent use of the best available evidence to guide policy decisions. It aims to ensure that policies are based on credible and relevant evidence while also considering stakeholder perspectives, equity, implementation feasibility, affordability, and sustainability ^1, 2^. Evidence can influence various stages of the policy process, including setting the agenda, shaping policy development, guiding implementation, and assessing outcomes through evaluation ^3^. Evidence-informed policymaking has gained increasing attention in recent years as governments and organisations worldwide recognise the importance of using high quality evidence to inform decision-making ^4^. The COVID-19 pandemic has further highlighted the critical role of evidence in guiding policy responses to complex societal challenges ^5^. In this context, there is a growing need to assess and strengthen the evidence-support ecosystems that underpin evidence-informed policymaking at both national and sub-national levels.

The Global Commission on Evidence to Address Societal Challenges (Global Evidence Commission) has been at the forefront of this movement, promoting the use of evidence to tackle societal challenges ^6^. They emphasised the need for robust national evidence infrastructures that encompass the research system, the evidence-support system, and the evidence-implementation system. They proposed that greater attention should be given to the evidence-support system, alongside a continuous focus on the evidence-implementation system, as both are essential for future efforts to effectively utilise evidence in addressing societal challenges ^6^. In January 2023, the Global Evidence Commission ^7^ released its first annual update, defining an evidence-support system as comprising three components:

- An evidence demand side focusing on how decision-makers use evidence, whether there is a culture that values evidence in policy processes, the capacity to use evidence and the enablers in place to support evidence use.
- Coordination mechanisms to help identify what evidence is needed on the supply side and to package the evidence in a way that decision-makers can use easily.
- An evidence supply of timely, demand-driven evidence from evidence-support units (either in-house or within partner organisations) for various forms of evidence, including data analytics, modelling, evaluation, behavioural and implementation research, qualitative insights, evidence synthesis, technology assessments, cost-effectiveness analysis, and guidance. This is further complemented by global evidence, such as living evidence syntheses.

The Global Evidence Commission has recommended that governments review their existing evidence-support systems to identify what is working well and could be scaled up, prioritise and address any gaps, and collaborate with policymakers, organisational leaders, professionals, and citizens to drive improvements ^6^. To support this review, they developed a tool called the Rapid Evidence Support System Assessment (RESSA) ^7, 8^, and they have engaged with partners in 12 countries to conduct RESSAs ^9^. The RESSA tool is licensed under a Creative Commons Attribution-NonCommercial-ShareAlike 4.0 International License. Copyright © 2024 McMaster University. Used with permission.

Building on the momentum generated by the Global Evidence Commission's reports, a significant collaboration has emerged in Ireland between the Health Research Board (HRB), the Department of Health, Evidence Synthesis Ireland (ESI) and Cochrane Ireland (based at the University of Galway), and the Global Evidence Commission. This collaboration aims to review and enhance the evidence-support system for health policymaking in Ireland, in accordance with the recommendations of the Global Evidence Commission ^6, 7^.

This paper outlines the methods utilised for the RESSA of health policymaking in Ireland. It elaborates on the methodology from the Global Evidence Commission ^7, 8^, providing a more comprehensive account of the involved process. A process evaluation is being conducted alongside the RESSA to assess its methodology and to refine and enhance the method for future evaluations ^10^.

## Aims and objectives

The RESSA project, led by the HRB, aims to evaluate Ireland's evidence-support system for health policymaking comprehensively. The objectives are to:

1. Examine the current state of Ireland’s evidence ecosystem for health policymaking, focusing on the demand and supply aspects of evidence usage and the coordination mechanisms between them.  
2. Identify effective strategies and components within the evidence-support system that currently function well for systematisation or scaling up. 
3. Evaluate the challenges, gaps, and barriers in the current evidence-support system, identifying and prioritising which areas to address to improve the overall decision-making processes. 
4. Engage with the RESSA Country Leads Group ^9^ to promote knowledge sharing, allowing Ireland to learn from international experiences and share its own lessons with other participating countries. 

## Methods

This study employs the RESSA approach, using websites, documents, and interviews to explore each of the three key components of an evidence-support system: the evidence demand and supply sides, and the coordination mechanism between them. This RESSA will utilise a flexible, mixed methods design, integrating qualitative and quantitative analyses. The study will be conducted in four main stages: 1. High-level website review; 2. In-depth website and document review; 3. Key stakeholder interviews; 4. Seeking feedback.

The project will be overseen by a dedicated oversight group convened and chaired by the HRB, comprising representatives from the Global Evidence Commission, the Department of Health, the Department of Further and Higher Education, Research, Innovation and Science (Evidence for Policy Unit), and ESI/Cochrane Ireland.

### 1. High-level website review

This stage will involve a systematic examination of relevant websites to identify key stakeholders, evidence-related activities, and publicly available resources within the evidence-support ecosystem. The review will focus on the organisational structure of the Department of Health to pinpoint key units and/or partners responsible for evidence support. It will provide the opportunity to review examples of evidence products from key stakeholders, such as the Health Information and Quality Authority (HIQA) and the HRB Evidence Centre.

### 2. In-depth website and document review

Building upon the initial website review, this stage will involve a more detailed analysis of the Department of Health website to gather information on how evidence support is approached from the evidence-demand side, the evidence-supply side, and the coordination mechanisms between both. Additionally, those participating in the key informant interviews will be asked to provide examples of documents that illustrate the evidence-support system, which were not available on the website. This stage will specifically address the first objective of the RESSA: to examine the current state of Ireland’s evidence ecosystem for health policymaking, focusing on the demand and supply aspects of evidence use and the coordination mechanisms between these. The overall synthesis of the findings will help identify successful strategies within the evidence-support system and the gaps and barriers as outlined in objectives 2 and 3.

***Search methods***

- Data sources include the Department of Health website, and key insights will also be gathered from key informant interviews.
- Search strategy: A search will be conducted using the following combinations of the keywords ‘evidence and research and policy’ and ‘evidence-informed policy.’

***Screening and selection***

Types of documents for inclusion:

- Strategies and frameworks for evidence use: Comprehensive strategic documents or frameworks that outline expectations, priorities, and approaches concerning the application of evidence in health policymaking.
- Documents on evidence support approaches: Any documents that describe or provide insights into how evidence support is approached, including methodological guidelines, standard operating procedures, or evidence-informed decision-making frameworks.
- Terms of reference or guidance for evidence production and support: Documents that outline the scope, objectives, and methodologies for creating evidence products, including guidelines for various forms of evidence synthesis.
- Committee terms of reference: Documents that define the roles, responsibilities, and operational procedures of committees or advisory groups engaged in evidence-informed decision-making processes within the health sector.
- Evaluations and assessments: Reports or documents that present the findings of evaluations or assessments conducted internally by organisations or as part of broader government requirements. These reports provide insights into the functioning, effectiveness, or impact of evidence support mechanisms.
- Evidence products: Examples (n=1-3) of reports, systematic reviews, guidelines, policy briefs, data analytics outputs, evaluations, or any other evidence outputs produced or commissioned by key stakeholders, such as the Department of Health, HRB, HIQA, and the Health Service Executive. Collecting examples of evidence products facilitates a manageable and focused analysis while still providing a representative overview of the types of evidence outputs. In deciding which documents to include, we will ensure representation of different types of evidence products and select documents with sufficient detail for analysis. We will also consider the publication dates of the documents and their impact and influence on policy decisions.
- Documents published in 2018 or later. The rationale for this time period is that Sláintecare, the Department of Health’s ten-year programme to transform health and social care services, was launched in 2018. Additionally, it encompasses documents produced in the two years before COVID-19 (2018 and 2019), the two years during the COVID-19 pandemic (2020 and 2021), and the two years following the peak of COVID-19 (2022 and 2023).

Types of documents for exclusion

- Documents lacking references to or illustrations of the evidence ecosystem regarding the demand and supply of evidence for health policymaking.
- Documents published prior to 2018.

***Document assessment***

Each document will be assessed to determine if it corresponds to any of the previously described document types and will be searched for the keywords “research”, “policy”, and “evidence”. Documents that seem to meet the inclusion criteria will be selected for full-text review. One researcher (BW) will perform the document search and initial screening.

Following the initial screening, the full texts of selected documents will be examined in detail to determine their eligibility for inclusion in the analysis. Documents that meet the inclusion criteria and offer relevant insights into the evidence-support ecosystem, insofar as they contain information extractable to answer questions in the data extraction framework (Appendix 1), will be included in the final analysis. Two researchers (BW and MT) will conduct the full-text screening. Initially, both researchers will review and discuss ten documents to ensure they share a mutual understanding of the inclusion and exclusion criteria. Once they are confident in their comprehension, they will independently screen an additional thirty documents. If disagreements arise, they will discuss the specific documents and present their rationales for inclusion or exclusion. If consensus cannot be reached, a third RESSA team member will serve as an arbitrator. If necessary, the researchers will refine the inclusion and exclusion criteria and review 10 additional documents together to ensure consistent application. Finally, they will separately screen the remaining documents before reconvening to compare and reconcile their findings. Any remaining discrepancies will be resolved through discussion and consensus, with the option to engage an arbitrator if needed. The screening and selection process will be documented using an adapted PRISMA (Preferred Reporting Items for Systematic Reviews and Meta-Analyses) flow diagram to ensure transparency ^11^.

***Data extraction, analysis and synthesis***

The data analysis and synthesis process will adhere to the READ approach ^12^, enabling a systematic method for organising, interpreting, and synthesising the information extracted from the selected documents. We will use the following four steps:

**Step 1: Ready materials**

A file naming system will be devised for the documents chosen for full text screening so that they can be easily retrieved throughout the research process.

**Step 2: Extract data**

The data extraction framework (Appendix 1) has been developed to meet the objectives of the document review stage, specifically to examine the current state of the evidence ecosystem for health policymaking by assessing both demand and supply aspects. The key variables to be extracted are informed by the relevant questions posed in a RESSA regarding the demand for evidence, the supply of evidence, and the interaction between both. A Word document with columns corresponding to the data extraction framework (Appendix 1) will be created. A sample of five documents representing the various types will be analysed using the data extraction framework. Two researchers (BW and MT) will analyse the documents separately, and any inconsistencies or difficulties encountered will be identified. The framework will be adjusted as necessary and finalised.

The relevant information from the documents will be summarised and entered into a Word document. This step will be performed by two researchers (BW and MT), with 20% of the documents cross-checked by each other.

**Steps 3 and 4: Analyse data and distil the findings**

Data analysis will begin during the data extraction phase, and memos will be written based on our observations and learnings from the data. Once data extraction is complete, the overall themes and patterns will be synthesised to provide an understanding of the Department of Health’s evidence-support ecosystem. This synthesis will consider successful strategies, barriers, and areas of opportunity within the system.

### 3. Key stakeholder interviews

 In-depth, semi-structured interviews with approximately 10 to 20 key stakeholders will be conducted. With the assistance of the HRB and the RESSA Oversight Group, these stakeholders will be strategically selected through purposive and snowball sampling to represent a diverse range of perspectives within the health policymaking evidence ecosystem. This includes policymakers from the Department of Health, evidence producers and researchers from both the Department of Health and external organisations such as HIQA and the HRB Evidence Centre, as well as individuals working at the intersection of research and policy. Those identified will be invited to participate in an individual or group interview conducted by a researcher from ESI. They will receive an information letter and have the opportunity to ask questions about the research. All interviewees will be required to sign a consent form.

Each interview will last approximately 45 to 60 minutes and will be conducted either in person or via video conferencing as an individual or group interview, depending on the availability and preference of the informants. The interview will begin with a brief presentation about the Global Evidence Commission and what is meant by the evidence-support system. The findings from the review of the websites and documents in previous steps will inform the development of interview questions, alongside more specific questions posed to interviewees in a RESSA ^8^ (examples of these questions are available in Appendix 2). Additionally, interviewees will be asked for any other documents that should be included in the document review, which shed light on the evidence-support system for health policymaking. The interviews will be audio recorded and transcribed. Data will be analysed using a thematic approach, employing a structured method such as the framework method ^13^, and NVivo V20 ^14^ will be used to facilitate data organisation and retrieval.

****Triangulation of data:**** As the document analysis and key informant interviews are expected to occur simultaneously, any additional documents identified during the interviews will be reviewed and incorporated into the analysis as appropriate. This iterative approach will facilitate the triangulation of findings from both data sources, thereby enhancing the robustness and credibility of the overall analysis.

### 4. Seek feedback

The oversight group will provide feedback on the main findings of the RESSA. Additionally, champions identified through the RESSA process and interviewed will be contacted by email for their input. This step will enable the team to refine its understanding of the evidence ecosystem.

## Ethical considerations

An application for the RESSA has been approved by the University of Galway Research Ethics Committee (Ref: 2024.01.003).

All participants will provide written informed consent before data collection. They will receive comprehensive information about the study, including its purpose, procedures, potential risks, and anticipated benefits. To ensure confidentiality, each participant will be assigned a unique identifier, and consent forms will be securely stored.

## Data management and privacy

All audio recordings from interviews will be deleted after transcription, and only anonymised transcripts will be retained. These transcripts, field notes, and documents will be stored for a minimum of seven years in accordance with the University of Galway Policy. In line with GDPR 2018 and the University of Galway Personal Data Security Schedule (PDSS), electronic records will be maintained on the University of Galway OneDrive server, which is accessed through a password-protected and encrypted laptop or desktop belonging to the research team. Consent forms will be stored either electronically in OneDrive or in a locked filing cabinet at the University of Galway. Individual names will not be linked to responses at any stage of the study. Access to data is restricted to the researchers involved in the project. If a professional transcription service is utilised, it will operate under stringent data confidentiality agreements.

## Dissemination

The results of the RESSA will be shared through publication in a peer-reviewed journal and presentations at national and/or international conferences. A presentation-style slide deck identifying gaps, barriers, and facilitators in the evidence-support system for health policymaking will also be prepared.

## Conclusion

In this paper, we present the methodology for a RESSA that will be conducted in Ireland on health policymaking. We expand on the methodology from the Global Evidence Commission ^7, 8^, offering a more detailed account of the process involved. While the original methodology provided an excellent foundation, our expanded approach aims to enhance the replicability and practical application of the methods by including additional details and explanations. Furthermore, the detailed methodology we present here facilitates easier comparison and contrast with similar assessments conducted by other groups. By outlining each step in greater depth, we aim to support a more consistent application of the RESSA across different contexts, enabling researchers and policymakers to draw clearer insights from cross-study comparisons.

While we initially focus on health policymaking, we anticipate that the project’s findings and methodologies will also be relevant to other government departments and cross-government evidence initiatives. This approach will aid in the evolution of Ireland's evidence-support system, providing valuable lessons and best practices that can enhance evidence-informed decision-making processes nationwide in areas such as climate change and education.

## Study status

The RESSA of health policymaking is in its final stages, and findings from this will be reported separately. By documenting the study design in this protocol, we aim to ensure transparency and promote reproducibility.

## Data Availability

No data are associated with this article Open Science Framework: A Rapid Evidence Support System Assessment (RESSA) of health policymaking in Ireland, https://doi.org/10.17605/OSF.IO/GKMDB ^15^. Extended data in this project pertains to the data extraction framework for the document review and the interview questions. This project contains the following referenced extended data: Appendix 1: Data Extraction Framework Appendix 2: Interview questions Data are available under the terms of the Creative Commons Attribution 4.0 International license (CC-BY 4.0).
