# Supplementary material for: A Rapid Evidence Support System Assessment (RESSA) of health policymaking in Ireland – A Protocol
Source: HRB Open Res. 2025 Jun 23;8:70. [Version 1] doi: 10.12688/hrbopenres.14167.1 (PMC12368487; doi:10.12688/hrbopenres.14167.1)
Supplement: Peer review history

# Reviewer report: Reviewer response for version 1

**DOI:** 10.21956/hrbopenres.15579.r48002

**Published:** 2025-08-20

**Authors:** Sarah Cusworth Walker

**Affiliations:** 1 University of Washington, Seattle, Washington, USA

Thank you for the opportunity to review this protocol paper. The study and paper are clearly explained. This purpose of the study is to use the RESSA approach to assess Ireland's evidence to policy ecosystem. As such, the research question being posed is whether Ireland has a sufficient evidence to policy ecosystem and the research approach is aimed at answering this question while providing guidance for addressing any identified gaps. 

 While the approach and rationale are clearly laid out, in the U.S. this might not be considered human subjects research as the purpose appears to be service improvement rather than drawing developing a generalizable conclusion or conducting research in support a larger program of research aimed at generalizable conclusions. To be a research study, again in the U.S., the protocol would identify questions that would allow the investigators to assess the appropriateness of the RESSA approach, itself, as a guide to evidence-informed policy making capacity. I offer this as an observation and possibly a suggestion for the authors to consider.

Is the study design appropriate for the research question?

Yes

Is the rationale for, and objectives of, the study clearly described?

Yes

Are sufficient details of the methods provided to allow replication by others?

Yes

Are the datasets clearly presented in a useable and accessible format?

Not applicable

Reviewer Expertise:

evidence informed policy, youth development and wellness, policy design, use of evidence, mental health policy

I confirm that I have read this submission and believe that I have an appropriate level of expertise to confirm that it is of an acceptable scientific standard.

# Reviewer report: Reviewer response for version 1

**DOI:** 10.21956/hrbopenres.15579.r47997

**Published:** 2025-07-08

**Authors:** Paul Cairney

**Affiliations:** 1 University of Stirling, Stirling, Scotland, UK

I welcome this study and wish the authors well. Therefore, all of the following points are for reflection, and I do not ask that the authors amend their protocol to address any of my quibbles.

 Some are minor points about the mild potential to confuse aspiration with reality when investigating the latter:

- ‘Evidence-informed policymaking’ is a vague term used by multiple authors to describe multiple things. If so, I suggest that the authors clarify that this is a specific (aspirational) definition, along the lines of ‘We describe/ treat evidence-informed policymaking as ..’
- The same could be said of ‘Evidence can influence various stages of the policy process’: this is a statement of aspiration, relating to simple models or official stories of policymaking. I suggest clarifying aims in relation to two different reference points: (1) what governments say they do or want to do (the stages, or *essential functions* or *requirements* of policymaking), versus (2) the policy processes that the authors seek to understand with this review (a messier picture in which an orderly or linear set of stages will not be so apparent).
- The metaphorical description of evidence-policy ‘ecosystems’ is handy for fans of brevity, but lacks meaning when investigating the details of policymaking and practice. Will this kind of term really guide any of the searches?

This point is slightly more substantive:

- Search methods. I suggest a more exploratory search before sticking with these terms, to check the extent to which government language is the same as researcher language. ‘Evidence-informed’ may not be the usual way to describe things, and ‘policy’ alone may not pick up all relevant activity.

These points are for serious reflection:

1. I think that the focus on ‘health’ is not as easy as it sounds, and could be too restrictive or misleading. I suspect that the ‘health’ focus will bias the research towards aspects such as healthcare, and not pick up on all relevant aspects of EIPM relating to the ‘wider’ or ‘social’ determinants of health. Indeed, if social determinants are so important, then the use of evidence in areas like social security, housing, education, etc. is just as relevant as in ‘health’. It would be worth describing a way to note and reflect on this limitation throughout the process (rather than simply note that other researchers can examine other sectors).
2. Asking people about their use of research is fine if you are interviewing health academics in government. However, I have found - in comparable research - that you restrict the research agenda too much by focusing so strongly on research use when interviewing policy actors. I suggest using a small number of exploratory interviews to ask more open questions about how research might fit into a wider policymaking context. For example, asking policy actors about the issues that command their attention, how they describe them, and what information they seek may be more fruitful than asking what health research evidence they use (at least until you establish that your frames of reference are sufficiently close).

Is the study design appropriate for the research question?

Yes

Is the rationale for, and objectives of, the study clearly described?

Yes

Are sufficient details of the methods provided to allow replication by others?

Yes

Are the datasets clearly presented in a useable and accessible format?

Yes

Reviewer Expertise:

Political science and policy studies

I confirm that I have read this submission and believe that I have an appropriate level of expertise to confirm that it is of an acceptable scientific standard.
